# Evaluating the diagnostic and prognostic utility of serum DLL1 in acute-on-chronic liver failure patients with bacterial infections

**DOI:** 10.3389/fmed.2026.1735014

**Published:** 2026-05-14

**Authors:** Juanjun Huang, Zhi Wang, Wei Zhu, Jian Chen

**Affiliations:** 1 Department of Infectious Diseases, Ganzhou Hospital-Nanfang Hospital, Southern Medical University (Ganzhou People's Hospital), Ganzhou, Jiangxi, China; 2 Department of Pathology, Ganzhou Hospital-Nanfang Hospital, Southern Medical University (Ganzhou People's Hospital), Ganzhou, Jiangxi, China; 3 Central Laboratory, Ganzhou Hospital-Nanfang Hospital, Southern Medical University (Ganzhou People's Hospital), Ganzhou, Jiangxi, China

**Keywords:** acute-on-chronic liver failure, bacterial infections, biomarker, delta-like ligand 1, diagnosis

## Abstract

**Background:**

The diagnosis of bacterial infections (BIs) in patients with acute-on-chronic liver failure (ACLF) remains a formidable clinical challenge, directly impacting mortality rates. Current biomarkers are often confounded by the profound systemic inflammation inherent to ACLF itself. The objective of this study was to evaluate the diagnostic and prognostic utility of serum Delta-like ligand 1 (DLL1) in ACLF patients with BIs.

**Methods:**

This retrospective diagnostic study utilized a pre-established, prospectively maintained ACLF cohort (*n* = 168). Serum DLL1 levels were measured with enzyme-linked immunosorbent assays, and diagnostic performance was assessed via receiver operating characteristic (ROC) curve analysis and multivariable regression.

**Results:**

In the ACLF cohort, serum DLL1 concentrations were significantly elevated in BI patients compared with their noninfected counterparts (*p* < 0.001). Multivariable regression revealed that DLL1 was an independent diagnostic predictor (adjusted OR = 1.982, 95% CI: 1.338–2.937). Moreover, the serum DLL1 level demonstrated substantial discriminative capacity (AUC = 0.815; 95% CI: 0.750–0.879), which improved when this variable was synergistically incorporated into a CRP-DLL1 dual-marker model (AUC = 0.852; *p* = 0.039 versus DLL1 alone). The stability of the combined model was further evaluated in subgroups with available procalcitonin (PCT) data. Decision curve analysis confirmed the clinical net benefit between probability thresholds from 10 to 70%. Crucially, the levels of DLL1 exhibited only marginal correlations with hepatic function parameters, indicating minimal confounding from liver inflammation, but failed to independently predict mortality in patients with ACLF and BI.

**Conclusion:**

Serum DLL1 represents a robust and clinically deployable diagnostic biomarker for BI in patients with ACLF, and its performance is minimally affected by underlying hepatic dysfunction. The synergistic CRP-DLL1 dual-marker model demonstrated enhanced diagnostic discrimination, significantly outperforming the individual biomarkers. Notably, the DLL1 level was not an independent predictor of mortality in this population with concurrent infection.

## 1 Introduction

ACLF is a critical syndrome characterized by rapid hepatic decompensation, multiorgan dysfunction, and high short-term mortality (1). Bacterial infection frequently triggers ACLF exacerbations and complicates clinical management; among cirrhotic patients with BI, 48% exhibit ACLF events that are etiologically linked to infectious causation (2). This susceptibility stems from profound immune paralysis, manifesting as impaired neutrophil phagocytosis, dendritic cell dysfunction, and dysregulated T-cell responses (3–5). Collectively, these conditions establish a permissive environment for pathogenic invasion. Infection-associated ACLF significantly worsens clinical complexity and prognosis (6).

Timely diagnosis remains challenging because of nonspecific clinical manifestations, low bacterial culture positivity rates, and rapid disease progression (7). Systemic inflammation affects all patients with ACLF, fundamentally compromising the reliability of conventional infection biomarkers, including CRP and PCT, in detecting BI (7, 8). Newly identified biomarkers, including presepsin, soluble triggering receptor expressed on myeloid cell-1 (sTREM-1), interleukin-6 (IL-6), and Golgi protein 73 (GP73), have shown promise but are inherently limited in their diagnostic performance (9–12). Current evidence highlights the limited discriminatory capacity of any single biomarker, necessitating urgent exploration of novel multivariable diagnostic approaches.

DLL1, a canonical transmembrane ligand within the Delta/Jagged family, initiates Notch signaling cascades upon receptor engagement. This protein controls essential biological processes including embryonic development, vascular morphogenesis, and hematopoietic differentiation (13). Critically, DLL1-mediated Notch signaling orchestrates immune cell functional specialization and effector responses, thereby influencing antimicrobial defense mechanisms (14–16). Exposure to bacterial pathogens or endotoxins rapidly activates DLL1-dependent pathways, driving amplified proinflammatory cascades that induce vascular endothelial dysfunction (17). Plasma concentrations of DLL1 have shown diagnostic utility across multiple sepsis cohorts, while recent translational studies have revealed its ability to predict postoperative infections in liver transplant recipients (18–21).

Although DLL1 demonstrates diagnostic capabilities in infection settings, its biomarker potential remains uncharacterized in patients with ACLF. This study therefore aims to bridge this gap through rigorous assessment of circulating DLL1 for BI identification within this critical patient population.

## 2 Materials and methods

### 2.1 Study population

This retrospective observational study enrolled patients from Ganzhou People’s Hospital between February 2023 and June 2024 who were derived from a prospective consecutively collected cohort, approved by the local ethics committee (trial code no. TY-ZKY2023-017-02). The original written informed consent explicitly permitted subsequent biomarker investigations using deidentified samples and data. The secondary analysis protocol developed for this study was given ethical approval by the Ganzhou People’s Hospital Ethics Committee (PJZKY2024-051-02). The study was conducted in accordance with the ethical principles of the Declaration of Helsinki (2024 revision), and its reporting conformed to the STARD guidelines (22). The inclusion criterion was a confirmed ACLF diagnosis, whereas the exclusion criteria comprised severe extrahepatic organ dysfunction other than liver-related complications, immunosuppressant use, organ transplantation history, hospitalization <24 h, or unavailable biospecimens.

### 2.2 Sample size calculation

The diagnostic cohort dimensions were prospectively established through statistical power analysis for biomarker validation studies. We predetermined that a minimum of 73 patients with BI and 73 noninfected controls would yield 95% confidence intervals with a maximum ±10% margin of error, predicated on a presumed diagnostic efficacy profile (sensitivity/specificity = 75%) derived from prior sepsis investigations (20), with a projected bacterial infection prevalence of 50% (7). Accounting for an anticipated 5% sample processing failure rate, this necessitated the enrollment of 154 evaluable subjects. Participant recruitment ultimately surpassed this threshold (*N* = 168), improving the analytical robustness of this study.

### 2.3 Clinical definitions

The diagnostic criteria adhered to those recommended in international guidelines. ACLF was diagnosed according to the Asian Pacific Association for the Study of the Liver (APASL) criteria as the presence of acute hepatic dysfunction (bilirubin ≥85 μmol/L, international normalized ratio (INR) ≥ 1.5) developing within 4 weeks in patients with chronic liver disease accompanied by ascites and/or encephalopathy (23). Acute kidney injury (AKI) was defined by the International Club of Ascites (ICA) consensus as a serum creatinine concentration ≥0.3 mg/dL within 48 h or a ≥ 50% increase from the baseline level (24).

BI were diagnosed according to the following composite clinical criteria. Spontaneous bacterial peritonitis (SBP) was diagnosed when the ascitic fluid neutrophil count was ≥250 cells/mm^3^, excluding secondary peritonitis. Urinary tract infections (UTIs) were defined by the presence of ≥1 symptom (fever [body temperature ≥38 °C], dysuria, urgency, frequency, or suprapubic pain) with a positive urine culture or the presence of ≥2 symptoms with pyuria (>10 leukocytes/μL). Pneumonia was diagnosed in accordance with radiographic evidence of new/progressive infiltrates plus either fever (body temperature ≥38 °C) or leukocytosis/leukopenia (12,000 or <4,000 cells/mm^3^) combined with respiratory symptoms (cough, purulent sputum, dyspnea, tachypnoea [heart rate >20/min], or abnormal auscultation) or microbiological confirmation from blood, pleural fluid, or Broncho alveolar lavage analysis. Spontaneous bacteremia was diagnosed on the basis of the presence of fever (body temperature ≥38 °C), chills, or hypotension with ≥2 positive blood cultures (skin contaminants excluded) and no identifiable infection source. The Centers for Disease Control (CDC) criteria were followed for the diagnoses of other infections (25). All cases were independently reviewed and confirmed by two senior attending physicians to ensure diagnostic accuracy.

### 2.4 Data collection and laboratory assays

Two independent clinicians extracted demographic, clinical, and laboratory parameters from our institutional electronic health records system. Peripheral blood samples obtained during initial clinical evaluations (≤24 h after admission) were immediately processed through refrigerated centrifugation (3,000 × g × 10 min, 4 °C) for serum isolation. Aliquoted serum was cryopreserved at −80 °C in barcoded vials until required for batch analysis. All serum samples were aliquoted upon initial storage. Each sample underwent only a single freeze–thaw cycle for its first to ensure pre-analytical uniformity. To maximize consistency, all DLL1 testing was performed in a batch within a narrow timeframe using the same lot of the commercial ELISA kit (Boster, EK1276, Wuhan), with all procedures strictly following the manufacturer’s instructions by a single experienced researcher. The test methodology involved the dilution of 50 μL of serum (1:2 v/v with assay diluent), 60 min of antigen–antibody conjugation at 37 °C, and spectrophotometric detection at 450 nm. Each sample was analyzed in duplicate per the kit specifications (calibrated range: 78–5,000 pg/mL; analytical sensitivity: 2 pg/mL; intra-assay CV < 8%). Concurrent laboratory tests (CRP, PCT, bilirubin, INR, and creatinine) were performed in the hospital’s accredited laboratory.

### 2.5 Statistical aanalysis

Continuous variables are expressed as the mean±SD or median [IQR] according to the data distribution (assessed with the Shapiro–Wilk test), whereas categorical data are reported as frequencies (%). Group differences were evaluated using the t test/Mann–Whitney U test for continuous variables and the χ^2^/Fisher’s exact test for categorical variables. BI predictors were identified through binary logistic regression: variables with *p* < 0.10 in the univariable analysis were entered into backward multivariable selection (retention *p* < 0.05). Diagnostic performance was assessed via ROC curve analysis to calculate the AUC with 95% CI (DeLong method) and Youden index-optimized thresholds, followed by decision curve analysis for determining clinical utility and calibration curve analyses. To address potential confounding bias, a propensity score matching (PSM) analysis was performed using the MatchIt package (version 4.5.5) in R, applying a 1:1 nearest-neighbor matching algorithm based on propensity scores estimated from a logistic regression model. Survival analyses were performed to address the impact of BI on 90-day mortality in patients with ACLF through Kaplan–Meier curve analyses and log-rank tests. For BI-related mortality in patients with ACLF, exploratory Cox modeling was conducted. Spearman correlations with Benjamini–Hochberg FDR adjustment were used to quantify biomarker–clinical parameter relationships. All analyses were performed in R4.2.1 (pROC, survival, survminer, ggplot2, rms and rmda packages), and a two-tailed *p* < 0.05 was considered to indicate statistical significance.

## 3 Results

### 3.1 Study population and baseline characteristics

Among 187 consecutively screened individuals with ACLF, 168 patients formed the final analytical cohort after the predefined eligibility criteria were implemented. This cohort demonstrated balanced stratification, with 84 confirmed BI cases and 84 noninfected controls (detailed screening attrition profile shown in Supplementary Figure S1).

**Figure 1:**
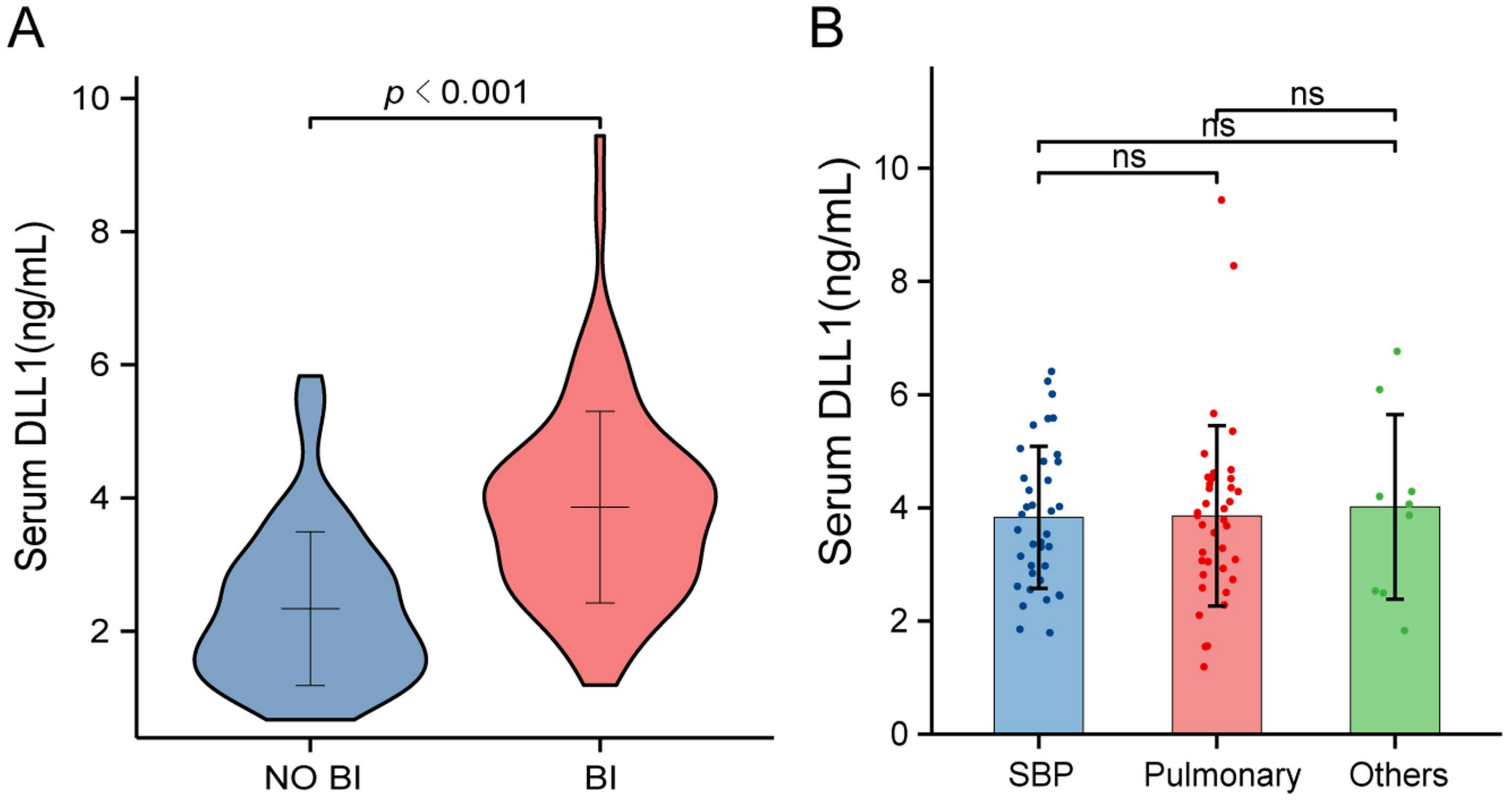
Discriminatory expression of DLL1 by infection status and sites in ACLF patients. **(A)** Serum DLL1 levels in BI versus NO BI controls. Compared with their NO BI counterparts, BI patients presented significantly elevated DLL1 levels (Mann–Whitney U test, *p* < 0.001). **(B)** DLL1 expression stratified by infection site category: SBP, pneumonia, and other infections. Group comparisons revealed no intertype differences (*Kruskal–Wallis test*, *p* = 0.972). BI, bacterial infection; DLL1, Delta-like ligand 1; SBP, spontaneous bacterial peritonitis.

BI patients exhibited significantly greater disease severity than their NO BI counterparts did, with higher prevalences of cirrhosis, ascites, and AKI; greater MELD-Na scores; elevated creatinine, CRP, PCT, and DLL1 levels and white blood cell (WBC) counts; and reduced albumin and sodium levels and platelet counts (all *p* < 0.05). The differences in the proportions of individuals with AKI (45.2% vs. 11.9%) and ascites (81.0% vs. 52.4%) were especially noteworthy. Age, sex distribution, etiology, and INR remained comparable (*p* > 0.05); detailed metrics are presented in Table 1. Serum DLL1 concentrations were significantly greater in BI patients than in NO BI controls (3.87 vs. 2.07 ng/mL, *p* < 0.001; Figure 1A), regardless of the site of infection (Figure 1B).

**Table 1:** Comparative clinical profiles stratified by bacterial infection status in ACLF.

Variables	NO BI (*n* = 84)	BI (*n* = 84)	*p value*
Sex, male	70 (83.3%)	64 (76.2%)	0.249
Age(years)	52 ± 12	54 ± 12	0.143
Etiology			0.480
HBV	54 (64.3%)	49 (58.3%)	
Alcohol	13 (15.5%)	20 (23.8%)	
Alcohol+HBV	8 (9.5%)	5 (6%)	
Others	9 (10.7%)	10 (11.9%)	
Cirrhosis	48 (57.1%)	67 (79.8%)	0.002**
MELD score	22 ± 5	26 ± 7	< 0.001***
MELD-Na score	23(19, 28)	28 (22, 38)	< 0.001***
Ascites	44 (52.4%)	68 (81.0%)	< 0.001***
HE	10 (11.9%)	19 (22.6%)	0.066
GI bleeding	2 (2.4%)	6 (7.1%)	0.277
AKI	10 (11.9%)	38 (45.2%)	< 0.001***
Total bilirubin (μmol/L)	195.0 (114.0, 352.6)	247.6 (142.1, 432.2)	0.055
Albumin (g/L)	31.5 ± 5.4	28.8 ± 5.0	0.001**
Creatinine (μmol/L)	68.0 (57.9, 80.0)	88.5 (69.9, 153.3)	< 0.001***
Sodium (mmol/L)	137.2 (134.2, 138.2)	134.4 (130.4, 137.0)	< 0.001***
INR	1.7 (1.5, 2.0)	1.8 (1.5, 2.2)	0.199
WBC count (×10^9^/L)	5.48 (4.06, 7.89)	7.95 (5.58, 12.40)	< 0.001***
Platelet count (×10^9^/L)	99 (68, 142)	75 (51, 120)	0.048*
CRP (mg/L)	13.1 (6.7, 25.1)	27.6 (18.4, 60.3)	< 0.001***
PCT (ng/mL)^†^	0.38 (0.23, 0.56)	0.79 (0.38, 3.15)	< 0.001***
DLL1 (ng/mL)	2.07(1.47, 2.93)	3.87 (2.84, 4.52)	< 0.001***

Data are expressed as mean ± SD (normally distributed variables), median [IQR] (non-normal distributions), or frequency (%) (categorical data). Statistical comparisons employed: Student’s *t*-test (parametric continuous variables), Mann–Whitney *U* test (nonparametric continuous), and *χ^2^*/Fisher’s exact test (categorical data, latter when expected cell counts <5). PCT^†^ measurements were available for a subgroup of 136 participants in the derivation cohort.*p < 0.05, **p < 0.01, ***p < 0.001. Alcohol+HBV indicates patients with both alcohol-related liver disease and hepatitis B virus infection. ACLF, Acute-on-chronic liver failure; BI, bacterial infection; NO BI, no bacterial infection; HBV, Hepatitis B virus; MELD, Model for end-stage liver disease; MELD-Na, Model for End-Stage Liver Disease sodium; HE, hepatic encephalopathy; GI, gastrointestinal; AKI, acute kidney injury; INR, international normalized ratio; WBC, white blood cell; CRP, C-reactive protein; PCT, procalcitonin; DLL1, Delta-like ligand.

### 3.2 Independent predictors of BI

Univariable analysis revealed that cirrhosis (OR = 2.956, 95% CI: 1.309–5.867), ascites (OR = 3.864, 95% CI: 1.933–7.724), total bilirubin (OR = 1.002, 95% CI: 1.000–1.004), albumin (OR = 0.905, 95% CI: 0.850–0.963), creatinine (OR = 1.020, 95% CI: 1.010–1.030), sodium (OR = 0.935, 95% CI: 0.884–0.989), WBC (OR = 1.144, 95% CI: 1.063–1.231), CRP (OR = 1.052, 95% CI: 1.029–1.075) and DLL1 (OR = 2.656, 95% CI: 1.914–3.687) were significant predictors of bacterial infection (all *p* < 0.05). Subsequent multivariable regression analysis revealed that cirrhosis (aOR = 4.705, 95% CI: 1.461–15.149; *p* = 0.009), CRP (aOR = 1.027, 95% CI: 1.004–1.052; *p* = 0.024) and DLL1 (aOR = 1.982, 95% CI: 1.338–2.937; *p* < 0.001) were independent predictors of BI, whereas ascites, bilirubin, albumin, creatinine, sodium and WBC were not (Table 2).

**Table 2:** Univariable and multivariable analysis of predictors of bacterial infection in ACLF.

Variables	Univariate analysis		Multivariate analysis	
	Odds ratio (95% CI)	*p value*	Odds ratio (95% CI)	*p value*
Sex, male	0.640 (0.299–1.372)	0.251	not included	
Age(years)	1.019 (0.994–1.045)	0.144	not included	
Etiology			not included	
HBV	Reference			
Alcohol	1.695 (0.763–3.766)	0.195		
Alcohol+HBV	0.689 (0.211–2.247)	0.537		
Others	1.224 (0.460–3.263)	0.685		
Cirrhosis	2.956 (1.489–5.867)	0.002**	4.705 (1.461–15.149)	0.009**
Ascites	3.864 (1.933–7.724)	< 0.001***	1.471 (0.536–4.036)	0.453
HE	2.163 (0.938–4.986)	0.070	1.292 (0.348–4.793)	0.702
GI bleeding	3.154 (0.618–16.098)	0.167	not included	
AKI	6.113 (2.780–13.440)	< 0.001***	0.761 (0.149–3.885)	0.743
Total bilirubin (μmol/L)	1.002 (1.000–1.004)	0.023*	1.002 (0.999–1.005)	0.189
Albumin (g/L)	0.905 (0.850–0.963)	0.002**	0.996 (0.902–1.100)	0.936
Creatinine (μmol/L)	1.020 (1.010–1.030)	< 0.001***	1.007 (0.992–1.022)	0.386
Sodium (mmol/L)	0.935 (0.884–0.989)	0.020*	0.960 (0.878–1.050)	0.371
INR	1.717 (0.903–3.264)	0.099	0.763 (0.288–2.025)	0.588
WBC count (×10^9^/L)	1.144 (1.063–1.231)	< 0.001***	1.113 (0.998–1.242)	0.055
Platelet count (×10^9^/L)	0.997 (0.992–1.002)	0.194	not included	
CRP (mg/L)	1.052 (1.029–1.075)	< 0.001***	1.027 (1.004–1.052)	0.024*
DLL1 (ng/mL)	2.656 (1.914–3.687)	< 0.001***	1.982 (1.338–2.937)	< 0.001***

*p < 0.05, **p < 0.01, ***p < 0.001. Alcohol+HBV indicates patients with both alcohol-related liver disease and hepatitis B virus infection. ACLF, Acute-on-chronic liver failure; HBV, Hepatitis B virus; HE, hepatic encephalopathy; GI, gastrointestinal; AKI, acute kidney injury; INR, international normalized ratio; WBC, white blood cell; CRP, C-reactive protein; DLL1, Delta-like ligand.

### 3.3 Diagnostic performance analysis

**Figure 2:**
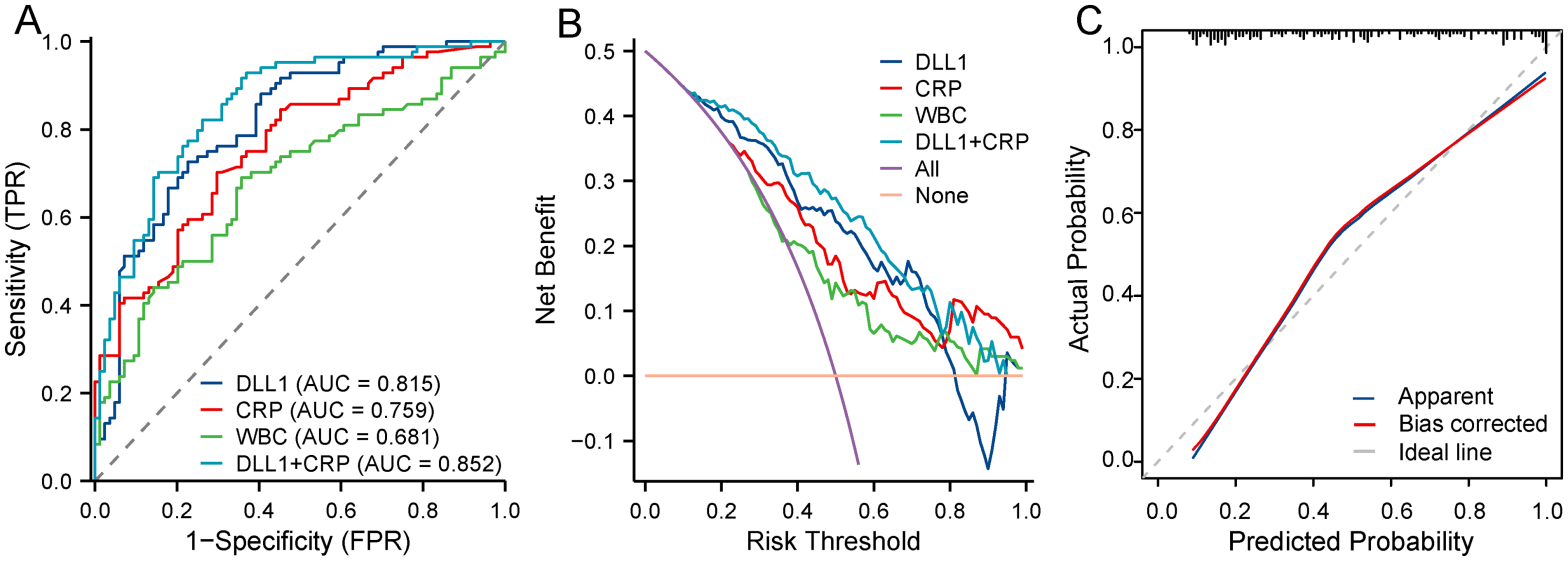
Diagnostic value of serum biomarkers for bacterial infection in ACLF. **(A)** ROC curve analysis demonstrating the comparative discriminability of the CRP, DLL1, WBC, and combined models. The results of the DeLong test were as follows: CRP vs. DLL1 (*p* = 0.198); CRP vs. combined (*p* = 0.002); WBC vs. combined (*p* < 0.001); and DLL1 vs. combined (*p* = 0.039). **(B)** Decision curve analysis showing greater net benefits of the combined model versus standalone biomarkers across 10–70% risk thresholds. **(C)** Calibration plot confirming accurate probability estimation (*Hosmer–Lemeshow p* = 0.376). The integrated approach significantly improved diagnostic precision through a combination of biomarkers. DLL1, Delta-like ligand 1; CRP, C-reactive protein; WBC, white blood cell; DCA, decision curve analysis; AUC, area under the curve.

Diagnostic performance analysis (Figure 2A) revealed that the combined CRP + DLL1 model achieved superior discrimination (AUC = 0.852, 95% CI: 0.794–0.909) over the individual biomarkers CRP (AUC = 0.759, 95% CI: 0.688–0.831; DeLong *p* = 0.002), DLL1 (AUC = 0.815, 95% CI: 0.750–0.879; *p* = 0.039), and WBC (AUC = 0.681, 95% CI: 0.599–0.762; *p* < 0.001). Among individual markers, DLL1 outperformed WBC (*p* = 0.010) but showed comparable discrimination to that of CRP (*p* = 0.198). At the optimal thresholds (Table 3), DLL1 exhibited the highest specificity (77.4%) among all the individually tested parameters, whereas the combined model provided substantially increased sensitivity (82.1%). The positive and negative predictive values (PPV and NPV) for DLL1 were 0.763 and 0.739, respectively, within this study cohort which had a BI prevalence of 50%.

**Table 3:** Diagnostic accuracy of serum biomarkers for bacterial infection in ACLF.

Model	AUC (95% CI)	Cut-off	Sensitivity	Specificity	PPV	NPV
DLL1 (ng/mL)	0.815 (0.750–0.879)	2.968	0.726	0.774	0.763	0.739
CRP (mg/L)	0.759 (0.688–0.831)	19.67	0.702	0.702	0.702	0.702
WBC (×10^9^/L)	0.681 (0.599–0.762)	6.115	0.690	0.643	0.659	0.675
Combined	0.852 (0.794–0.909)	0.436	0.821	0.738	0.758	0.805

Combined model: logistic regression integrating DLL1 and CRP levels.

ACLF, Acute-on-chronic liver failure; DLL1, Delta-like ligand 1; CRP, C-reactive protein; WBC, white blood cell; PPV, positive predictive value; NPV, negative predictive value.

Decision curve analysis (Figure 2B) demonstrated consistently superior clinical utility for the combined model over standalone biomarkers (CRP, WBC, and DLL1), delivering greater net benefit across clinically relevant threshold probabilities (10–70%). The calibration assessment (Figure 2C) confirmed the reliability of the concordance between the predicted and observed outcomes (Hosmer–Lemeshow *p* = 0.376).

**Figure 3:**
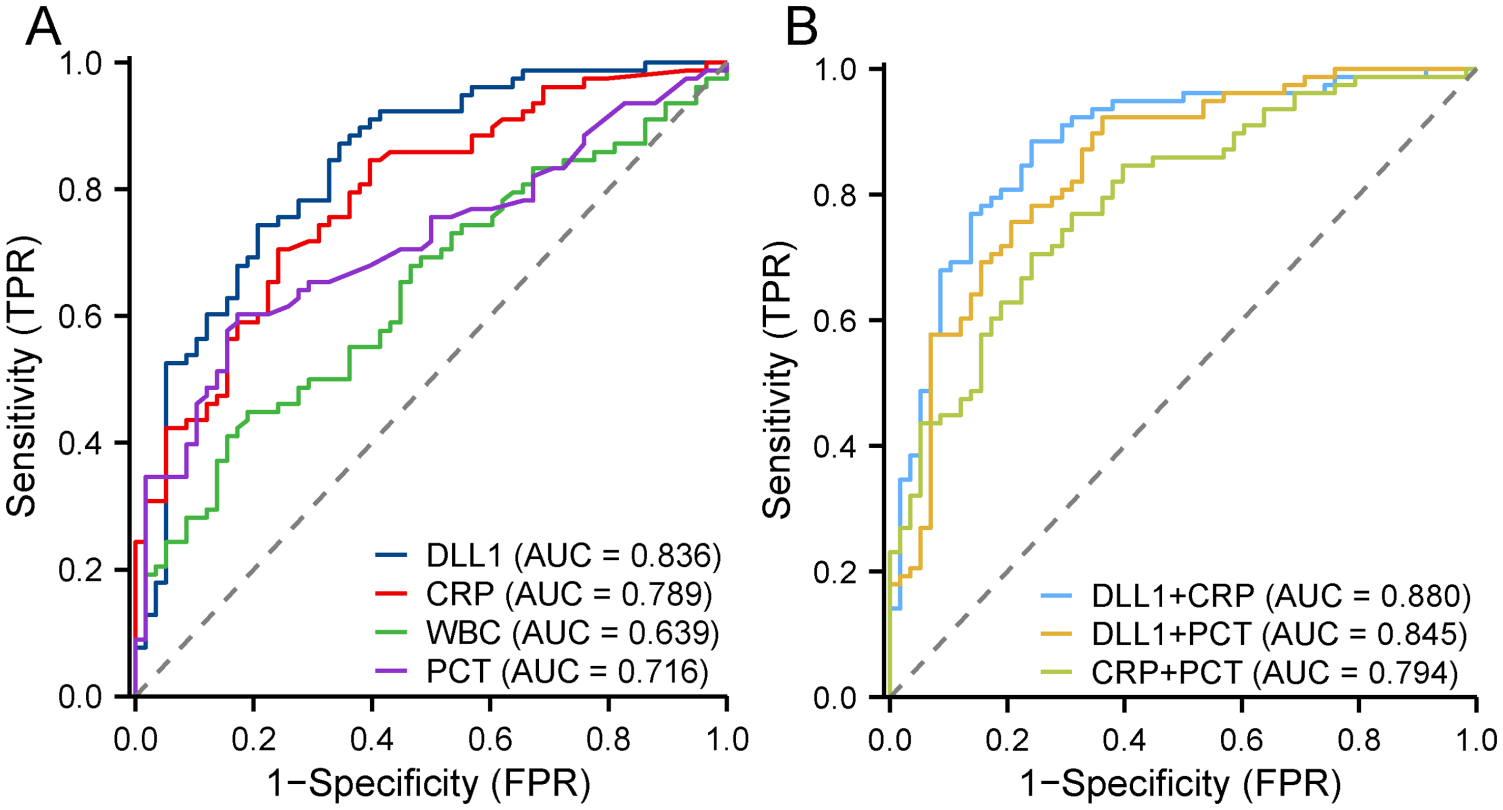
Diagnostic utility of serum biomarkers in the subgroup of participants with available PCT data (*n* = 136). **(A)** Discriminatory performance: DLL1 (AUC = 0.836) outperformed PCT (AUC = 0.716; *p* = 0.023) and WBC (AUC = 0.639; *p* < 0.001) but was comparable in performance to CRP (AUC = 0.789; *p* = 0.309). **(B)** The CRP-DLL1 model was superior to the models involving other dual-marker combinations and standalone biomarkers (ΔAUC +0.035–0.241; DeLong *p* < 0.05 for all comp arisons). PCT, procalcitonin; DLL1, Delta-like ligand 1; CRP, C-reactive protein; WBC, white blood cell.

Within the PCT-available cohort (*n* = 136/168, 81.0%, PCT testing was performed based on real-world clinical indications (typically in cases with more severe illness or suspected infection); Figure 3A), serum DLL1 demonstrated a significantly superior discriminatory capacity (AUC = 0.836, 95% CI: 0.767–0.906) compared with WBC (AUC = 0.639; *p* < 0.001) and PCT (AUC = 0.716; *p* = 0.023) but maintained a statistically comparable performance to that of CRP (AUC = 0.789; *p* = 0.309). The CRP-DLL1 dual-marker model exhibited enhanced diagnostic accuracy compared with the other comparator approaches—including CRP-PCT, DLL1-PCT, and standalone biomarker models (CRP/WBC/PCT/DLL1)—with statistically significant improvements across all pairwise comparisons (DeLong’s *p* < 0.05; Figure 3B). The subgroup with PCT data exhibited significantly higher disease severity, including elevated MELD-Na, creatinine, and INR levels, compared to patients without PCT data (all *p* < 0.05, Supplementary Table S1).

To address potential baseline imbalances between the BI and non-BI groups that might confound the discriminatory performance of DLL1, we conducted PSM. Using a 1:1 nearest-neighbor algorithm (spacing = 0.1, no-replacement), we matched patients on three key covariates: MELD-Na, creatinine, and AKI status. This resulted in 49 successfully matched pairs. After matching, there were no significant differences in MELD-Na, creatinine, or AKI status between the groups (all *p* > 0.05), and the standardized mean differences (SMD) for all three covariates were below 0.1, indicating substantially improved balance (Supplementary Table S2). In this matched cohort, serum DLL1 levels remained significantly higher in the BI group compared to the non-BI group (3.53 ng/mL vs. 2.10 ng/mL, *p* < 0.001). We then performed univariable and multivariable logistic regression analyses incorporating all baseline variables from the matched cohort. In the final multivariable model, both cirrhosis and serum DLL1 level emerged as independent predictors of BI. DLL1 was significantly associated with BI (OR: 1.722 per ng/mL, 95% CI: 1.086–2.730; *p* = 0.021; Supplementary Table S3). In the PSM cohort (*n* = 98), the diagnostic performance of DLL1 was evaluated and compared to conventional biomarkers. The AUC for DLL1, CRP, and WBC was 0.754, 0.651, and 0.597, respectively. DLL1 demonstrated superior diagnostic accuracy compared to WBC (*p* = 0.030) and comparable performance to CRP (*p* = 0.123; Supplementary Figure S2).

### 3.4 Correlations between DLL1 levels and clinical parameters

**Figure 4:**
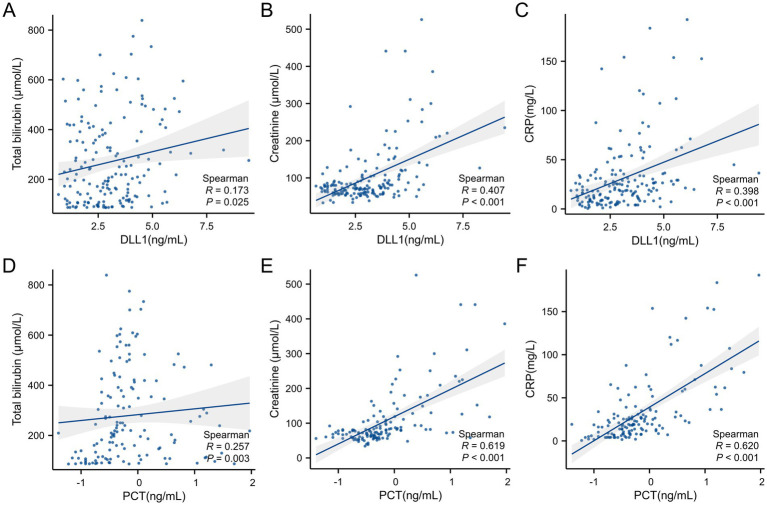
Biomarker–clinical parameter correlations. Notes: For improved visualization in panels (D–F), PCT values are displayed on a logarithmic scale (log₁₀PCT), while statistical correlations (*ρ* and *p* values) remain based on raw PCT concentrations. This transformation does not affect correlation coefficients or significance levels. **(A)** DLL1-total bilirubin (*ρ* = 0.173; *p* < 0.05), **(B)** DLL1-creatinine (*ρ* = 0.407; *p* < 0.001), **(C)** DLL1-CRP (*ρ* = 0.398; *p* < 0.001), **(D)** PCT-total bilirubin (*ρ* = 0.257; *p* < 0.05), **(E)** PCT-creatinine (*ρ* = 0.619; *p* < 0.001), **(F)** PCT-CRP (*ρ* = 0.620; *p* < 0.001). DLL1, Delta-like ligand 1; CRP, C-reactive protein; PCT, procalcitonin.

The data in Supplementary Table S4 and Figure 4 indicate that serum DLL1 expression was weakly correlated (|*ρ*| < 0.30; *p* < 0.05) with total bilirubin, albumin, INR, and white blood cell count. Moderately positive associations were observed with the MELD score (*ρ* = 0.404), MELD-Na score (0.392), creatinine (0.407), CRP (0.398), and procalcitonin (0.385) (all *p* < 0.001). Serum PCT was weakly correlated with total bilirubin (*ρ* = 0.257; *p* < 0.05) but strongly correlated with creatinine (0.619) and CRP (0.620) (both *p* < 0.001).

### 3.5 Prognostic analysis

Compared with that of their NO BI counterparts, the 90-day survival of BI patients was substantially impaired (40% vs. 70%; log-rank *p* < 0.001) (Supplementary Figure S3). Mortality was predominant within the initial 30-day period, underscoring the critical prognostic significance of BI.

In the prognostic analysis of 90-day mortality in patients with ACLF and BI, univariable Cox regression revealed hepatic encephalopathy (HR = 5.589, 95% CI: 3.036–10.291), gastrointestinal bleeding (HR = 2.785, 95% CI: 1.102–7.038), total bilirubin (HR = 1.002, 1.001–1.004), creatinine (HR = 1.005, 95% CI: 1.003–1.007), INR (HR = 3.459, 95% CI: 2.252–5.313), WBC (HR = 1.020, 95% CI: 1.003–1.038), and serum DLL1 (HR = 1.267, 95% CI: 1.060–1.514) as significant predictors (all *p* < 0.05). Multivariable analysis confirmed independent associations for hepatic encephalopathy (aHR = 4.058, 95% CI: 1.905–8.645; *p* < 0.001), gastrointestinal bleeding (aHR = 3.492, 95% CI: 1.261–9.668; *p* = 0.016), total bilirubin (aHR = 1.002, 95% CI: 1.000–1.004; *p* = 0.029), and INR (aHR = 1.974, 95% CI: 1.129–3.452; *p* = 0.017), while the associations for creatinine, WBC, and DLL1 were no longer significant (Table 4).

**Table 4:** Univariable and multivariable cox regression analysis of 90-day mortality predictors in ACLF patients with bacterial infection.

Characteristics	Univariate analysis		Multivariate analysis	
	Hazard ratio (95% CI)	*p value*	Hazard ratio (95% CI)	*p value*
Sex, male	1.368 (0.737–2.539)	0.321	not included	
Age (years)	1.018 (0.994–1.043)	0.143	not included	
Etiology			not included	
HBV	Reference			
Alcohol	0.980 (0.501–1.915)	0.953		
Alcohol+HBV	0.219 (0.030–1.608)	0.135		
Others	0.989 (0.434–2.254)	0.980		
Cirrhosis	0.806 (0.412–1.574)	0.527	not included	
Ascites	1.151 (0.560–2.369)	0.702	not included	
HE	5.589 (3.036–10.291)	< 0.001***	4.058 (1.905–8.645)	< 0.001***
GI bleeding	2.785 (1.102–7.038)	0.030*	3.492 (1.261–9.668)	0.016*
AKI	2.970 (1.678–5.257)	< 0.001***	1.486 (0.664–3.325)	0.336
Total bilirubin (μmol/L)	1.002 (1.001–1.004)	0.001**	1.002 (1.000–1.004)	0.029*
Albumin (g/L)	0.975 (0.917–1.037)	0.425	not included	
Creatinine (μmol/L)	1.005 (1.003–1.007)	< 0.001***	1.002 (0.998–1.006)	0.319
Sodium (mmol/L)	1.031 (0.988–1.077)	0.162	not included	
INR	3.459 (2.252–5.313)	< 0.001***	1.974 (1.129–3.452)	0.017*
WBC (×10^9^/L)	1.020 (1.003–1.038)	0.022*	1.021 (0.998–1.044)	0.071
Platelet (×10^9^/L)	0.999 (0.994–1.004)	0.654	not included	
CRP (mg/L)	1.004 (0.998–1.010)	0.161	not included	
DLL1 (ng/mL)	1.267 (1.060–1.514)	0.009**	1.064 (0.840–1.348)	0.607

*p < 0.05, **p < 0.01, ***p < 0.001. Alcohol+HBV indicates patients with both alcohol-related liver disease and hepatitis B virus infection. ACLF, Acute-on-Chronic Liver Failure; HBV, Hepatitis B virus; HE, hepatic encephalopathy; GI, gastrointestinal; AKI, acute kidney injury; INR, international normalized ratio; WBC, white blood cell; CRP, C-reactive protein; DLL1, Delta-like ligand 1.

## 4 Discussion

Patients with ACLF exhibit exceptionally high rates of BI, with a global prevalence exceeding 50% (7). Notable epidemiological variations exist across regions: in Chinese cohorts, hepatitis B virus (HBV) reactivation and BI are the primary ACLF triggers, whereas European populations frequently develop ACLF following severe BI or alcohol-related hepatitis (2, 26). Critically, multidrug-resistant (MDR) organisms represent a major concern, with prospective studies confirming that MDR infections independently predict mortality (adjusted OR = 4.41) through direct effects on disease progression and therapeutic constraints (27, 28). Consequently, identifying new biomarkers for rapid identification of BI in ACLF cohorts is crucial. The ideal biomarker should not only signal the presence of infection but also possess sufficient specificity to help avoid unnecessary empiric antibiotic therapy, a key consideration in mitigating MDR selection pressure. DLL1 emerges as a candidate that may meet these criteria. DLL1 has been established as a diagnostic and prognostic biomarker in sepsis across multiple clinical cohorts (18, 20, 29). Building upon our prior findings that established DLL1 as a robust diagnostic biomarker for BI in decompensated cirrhosis, this study extends the investigation to ACLF cohorts (30). ACLF is a distinct entity characterized by systemic hyperinflammation and multiorgan failure (31). This study revealed five key findings: (a) serum DLL1 levels were significantly higher in patients with ACLF and BI than in uninfected controls regardless of the site of infection; (b) elevated DLL1 levels independently predicted BI; (c) DLL1 showed favorable diagnostic performance for BI (AUC = 0.815), at its optimal threshold, DLL1 demonstrated a higher specificity than both CRP and WBC, while the combination model (CRP-DLL1) provided greater discriminability according to decision curve analysis, confirming its clinical applicability; (d) only a weak correlation was observed between DLL1 and liver inflammation biomarkers; and (e) DLL1 levels did not independently predict the outcomes of patients with ACLF and concurrent infections.

Consistent with patients with infection-associated pathophysiological burdens, patients with ACLF and BI exhibited significantly greater disease severity than their uninfected counterparts did. These individuals demonstrated substantially higher frequencies of cirrhosis complications (ascites and AKI), along with elevated MELD-Na scores, creatinine, and inflammatory markers, including WBC, CRP, and PCT. Concurrently, significantly reduced albumin, sodium, and platelet levels were observed in infected patients. These clinical features align consistently with those of previously published studies (9, 32). Crucially, serum DLL1 concentrations were markedly higher in infected patients (3.87 ng/mL vs. 2.07 ng/mL, *p* < 0.001), maintaining consistent levels across diverse infection sites. Following multivariable adjustment, only cirrhosis (aOR = 4.71), CRP (aOR = 1.03) and DLL1 (aOR = 1.98) were robust independent predictors following covariate adjustment, whereas conventional hepatic and renal parameters (ascites, bilirubin, albumin, creatinine) along with sodium and WBC lost statistical significance. These findings underscore the imperative for heightened clinical vigilance toward BI in ACLF patients with preexisting cirrhosis, necessitating rigorous surveillance protocols for prompt detection and immediate therapeutic intervention without delay.

CRP and PCT remain adopted biomarkers for BI detection in patients with decompensated cirrhosis, demonstrating modest diagnostic accuracy within this population (33). However, this conventional utility becomes significantly compromised in patients with ACLF because of three constraints: (a) systemic inflammation universally affects patients with ACLF irrespective of infection status; (b) increased CRP levels exhibit poor specificity through indiscriminate responses to diverse inflammatory triggers, including viral infections, sterile inflammation, and tissue necrosis; and (c) emerging evidence calls the diagnostic reliability of PCT in liver failure contexts into question due to interference from profound hepatic inflammation and necrosis (8, 31, 34). Renal impairment-mediated PCT elevations in patients with AKI intrinsically impair its diagnostic reliability for bacterial infections, necessitating threshold recalibration to maintain clinical utility (35). Critically, our cohort demonstrated both a high prevalence of AKI (45.2%) and significant renal dependence of procalcitonin, with a strong correlation between the serum PCT concentration and creatinine level (*ρ* = 0.619, *p* < 0.001). Consequently, evidence-based interpretive thresholds for PCT in patients with ACLF-associated infections remain clinically undefined. In our study, diagnostic evaluation revealed that the integrated CRP-DLL1 model achieved an AUC of 0.852, significantly outperforming individual biomarkers through pairwise comparisons (CRP, WBC and DLL1). Although CRP and DLL1 demonstrated comparable diagnostic performance (*p* = 0.198), DLL1 provided maximal specificity (77.4%) at the established thresholds. Decision curve analysis confirmed that the combination model provided enhanced clinical utility across relevant risk thresholds (10%–70%), generating significantly greater net benefit than any standalone approach. Crucially, in the subgroup of participants with available PCT data, DLL1 maintained robust performance comparable to that of CRP but reliably surpassed both PCT and WBC. The CRP-DLL1 combination further demonstrated a quantifiable advantage over alternative dual-marker constructs, including CRP-PCT and DLL1-PCT, with statistically confirmed improvements in classification accuracy across all comparative analyses. It should be noted that the subgroup with available PCT data had significantly higher baseline disease severity. The diagnostic performance of the combined model remained robust when further evaluated in a sensitivity analysis of this subgroup with available PCT data. To address potential confounding, a PSM was conducted. The key finding is that after balancing key covariates, DLL1’s association with BI remained significant, both as an independent predictor and a diagnostic biomarker. This consistency strengthens the robustness of our primary results. Nonetheless, the matched cohort had a reduced sample size, which may introduce bias. Therefore, while this analysis provides valuable supplementary evidence, the definitive utility of DLL1 requires further validation in larger prospective studies.

Our analysis demonstrated substantially impaired 90-day survival in ACLF patients with BI compared with their NO BI counterparts (40% vs. 70%), aligning with established evidence linking BI to accelerated mortality in patients with hepatic failure (12). Importantly, this critical prognostic significance peaked within the initial 30-day period, indicating both a distinct mortality pattern and that early infection control may fundamentally alter clinical outcomes. Prognostic factor analysis revealed hepatic encephalopathy, gastrointestinal bleeding, elevated bilirubin, and prolonged INR as independent predictors of 90-day mortality in patients with ACLF and BI, underscoring that the high mortality in these patients is predominantly driven by severe hepatocellular injury coupled with devastating complications. Although univariable analysis revealed a significant association for serum DLL1 (HR = 1.27), its prognostic effect became nonsignificant following multivariable adjustment. Therefore, serum DLL1 can serve as a diagnostic biomarker for BI in patients with ACLF but may not be an independent predictor of mortality. We acknowledge that the exploratory prognostic analysis of DLL1 may be influenced by unmeasured variables, particularly those related to infection management and microbiology (e.g., timing of antibiotic therapy, prevalence of multidrug-resistant pathogens).

Recent evidence substantiates that DLL1 is diagnostically superior to conventional biomarkers, including CRP, PCT, and WBC, in identifying sepsis (19). The characteristic dynamic elevation pattern of this biomarker has been reported to feature rapid escalation during the early sepsis phase followed by maintenance of this level through day 7 (20). Further substantiating these observations, Schneck et al. (18) specifically demonstrated that septic shock patients exhibited significantly elevated DLL1 concentrations compared with abdominal surgery controls and validated its prognostic capacity in AKI risk stratification. Registry analyses have established three cardinal associations: (1) the degree of increase in DLL1 is correlated with infection foci (abdominal infections, primary bacteremia, surgical site infections); (2) significantly greater elevations accompany gram-negative versus gram-positive infections; and (3) DLL1 independently predicts mortality in patients with sepsis (29). This study identifies DLL1 as a novel diagnostic biomarker for BI in patients with ACLF, with the integrated CRP-DLL1 model demonstrating significantly enhanced diagnostic accuracy. Notably, DLL1 concentrations exhibited no statistically significant variations across distinct infection sites within our cohort, nor did DLL1 emerge as an independent predictor of mortality in this population, contrasting sharply with established evidence for sepsis. The observed discrepancies from established sepsis studies likely reflect the compounded pathological burden in ACLF, wherein ACLF-induced immunological paralysis and depleted microbial richness collectively confer distinctive infection biomarker behavior compared with severe non-ACLF infections (4, 36). Mortality in patients with ACLF and BI remains independently predicted by indicators of hepatocellular injury severity (total bilirubin and INR) rather than conventional infection markers such as CRP or PCT, confirming that the hepatic necroinflammatory burden supersedes infectious parameters in determining clinical outcomes (37).

Mechanistic insights revealing the persistently weak correlations of DLL1 with liver function indices (total bilirubin, albumin, and INR; all |*ρ*| < 0.30) substantiate its ability to function through pathway-insulated signaling mechanisms, maintaining diagnostic independence from hepatic inflammatory interference. While demonstrating a moderate correlation with CRP (*ρ* = 0.398), DLL1 retains an independent predictive capacity for BI. Critically, the combined CRP-DLL1 model achieves synergistic diagnostic enhancement through orthogonal pathogen recognition mechanisms. By functioning as a sentinel regulator of the Notch cascade, DLL1 governs adaptive immunity through precise coordination of immune cell differentiation and effector programming (38). This regulatory architecture is pathologically exaggerated during inflammatory states, ultimately dictating the amplitude of the systemic response and kinetics of progression (39, 40). Substantial evidence has verified the ability of DLL1 to dynamically modulate endothelial barrier integrity and inflammation-driven vascular permeability via canonical Notch transduction (17, 41).

Several methodological limitations in this investigation should be acknowledged. Our study enrolled a predominantly HBV-related ACLF cohort using APASL criteria, this specific etiological and diagnostic framework may limit the direct extrapolation of our findings to ACLF populations with different causes or diagnosed under other criteria, such as EASL-CLIF. The monocentric design and statistical power restrictions necessitate cautious interpretation. The temporal trajectory and therapeutically modulated dynamics of DLL1 (e.g., changes following antibiotic therapy) remain uncharted, limiting our ability to assess its utility in monitoring treatment response. Although subgroup analyses addressed biases from partial procalcitonin data, residual confounding from unmeasured covariates persists as an inherent limitation. The PPV and NPV for DLL1 were calculated based on the 50% prevalence of BI in our matched cohort, these predictive values may not be directly generalizable to clinical settings with a different pre-test probability of infection. Due to its retrospective design, this study lacks formal stability validation for the DLL1 assay and its reported absolute values should not be directly compared with those from different platforms or sample types; future prospective studies with method standardization are required before clinical application. The prognostic analysis for DLL1 is limited by the retrospective design, not all potential confounders could be adjusted for, and the null association with mortality may be masked by unmeasured treatment and pathogen-related factors. Targeted experimental validation is imperative for establishing mechanistic causality between DLL1 and BI in patients with ACLF.

## 5 Conclusion

Serum DLL1 is a reliable diagnostic biomarker for BI in patients with ACLF and its performance is minimally influenced by hepatic dysfunction. The combined CRP-DLL1 model demonstrates superior diagnostic performance over individual biomarkers. Despite lacking prognostic value for mortality, this model enables timely infection detection for guiding early antimicrobial therapy in the management of ACLF.

## Data Availability

The raw data supporting the conclusions of this article will be made available by the authors, without undue reservation.
