# Supplementary figures and images for: Evaluating the diagnostic and prognostic utility of serum DLL1 in acute-on-chronic liver failure patients with bacterial infections
Source: Front Med (Lausanne). 2026 May 14;13:1735014. doi: 10.3389/fmed.2026.1735014 (PMC13215816; doi:10.3389/fmed.2026.1735014)

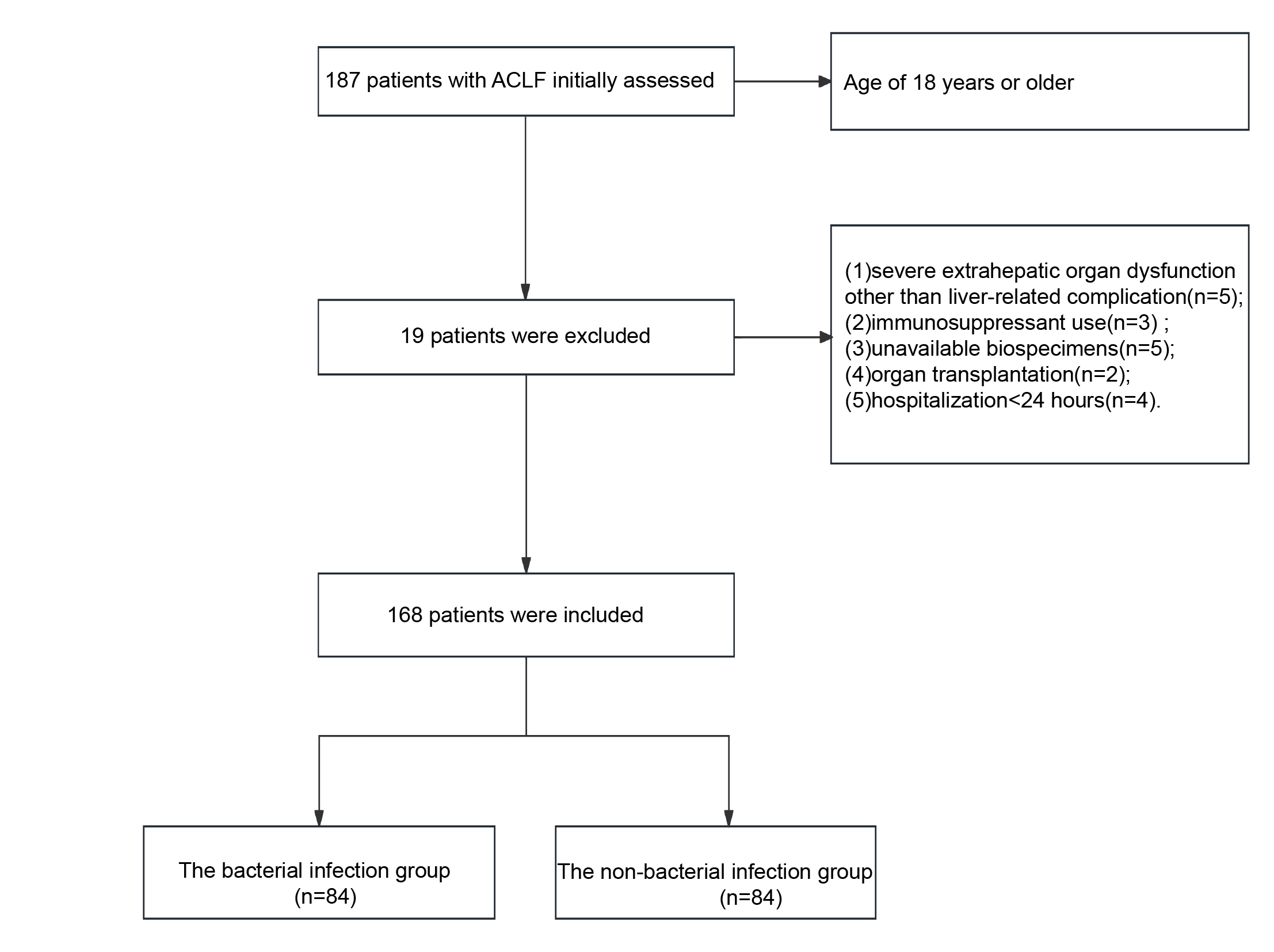

Supplement: Supplementary file 1 [file Image_1.tif]

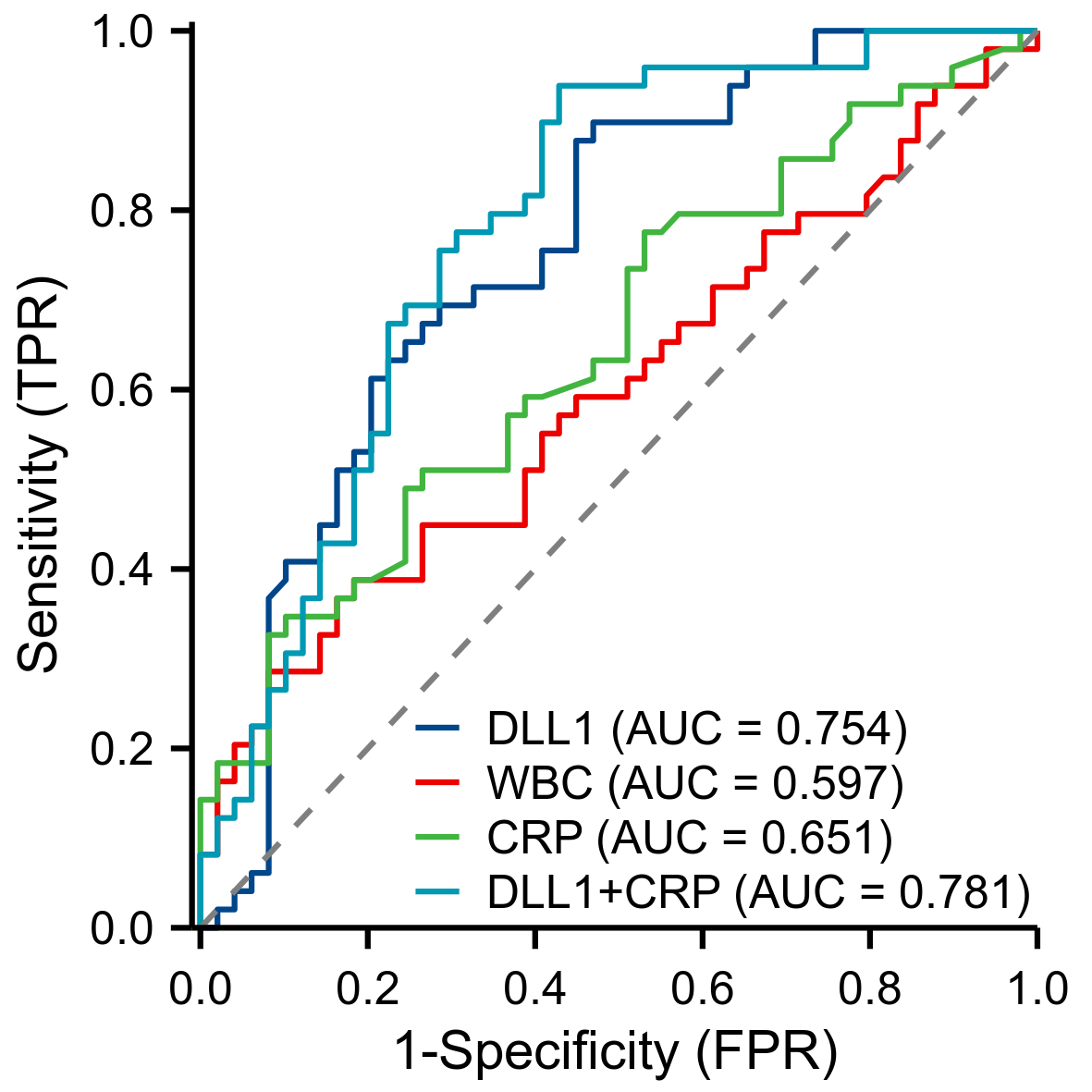

Supplement: Supplementary file 2 [file Image_2.tiff]

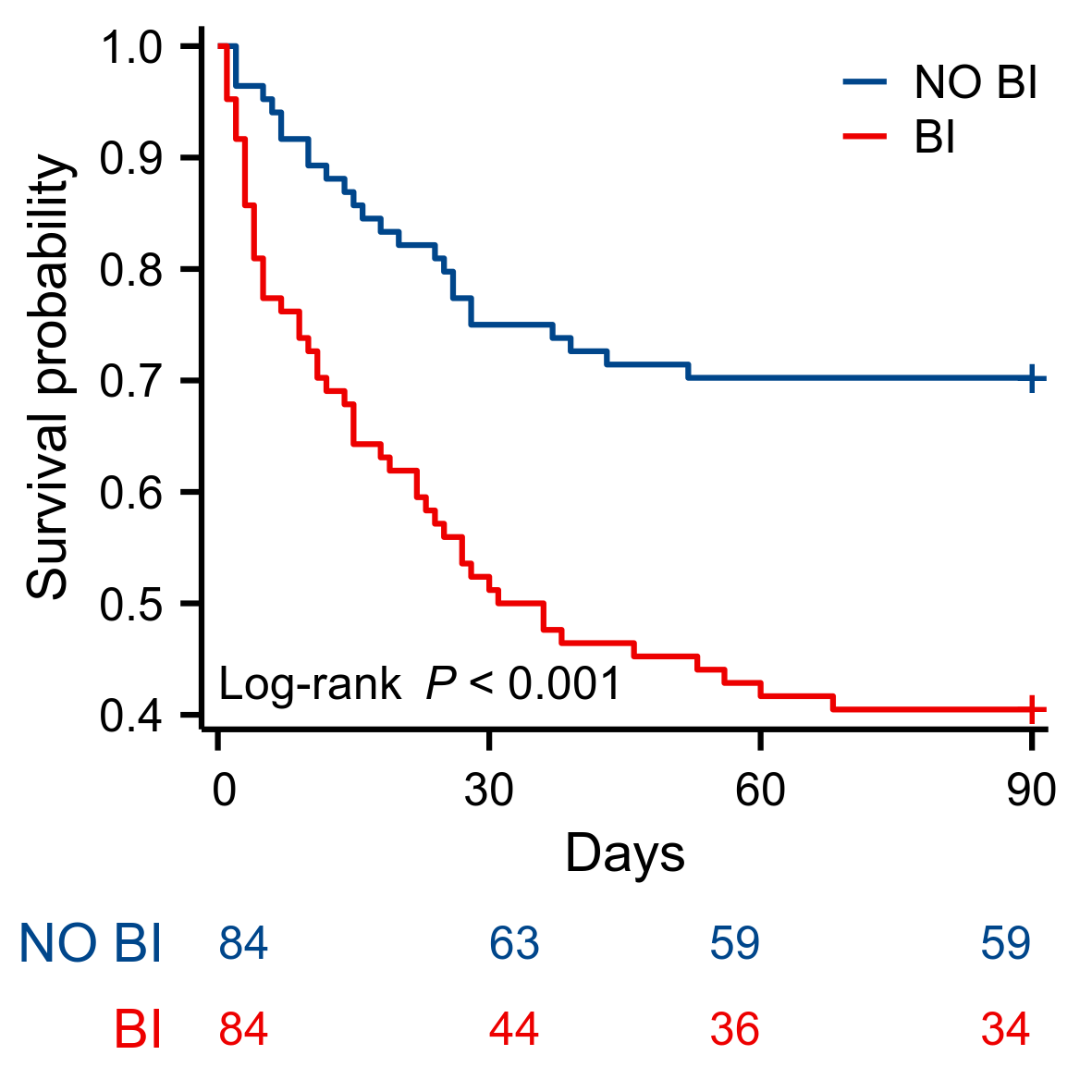

Supplement: Supplementary file 3 [file Image_3.tiff]
