# Supplementary material for: Evaluating the diagnostic and prognostic utility of serum DLL1 in acute-on-chronic liver failure patients with bacterial infections
Source: Front Med (Lausanne). 2026 May 14;13:1735014. doi: 10.3389/fmed.2026.1735014 (PMC13215816; doi:10.3389/fmed.2026.1735014)
Supplement: Supplementary file 4 [file Supplementary_file_1.pdf]

SUPPLEMENTAL FIGURE LEGENDS

SUPPLEMENTARY FIGURE 1. Participant Selection Workflow. Schematic of the screening methodology, application of exclusion criteria, and final cohort inclusion pathways for the ACLF population.

SUPPLEMENTARY FIGURE 2. Diagnostic Performance of Serum Biomarkers in the PSM Cohort (n=98). The AUC for DLL1, CRP, and WBC was 0.754, 0.651, and 0.597, respectively. DLL1 demonstrated superior diagnostic accuracy compared to WBC (P=0.030) and comparable performance to CRP (P=0.123). DLL1, delta-like ligand 1; CRP, C-reactive protein; WBC, white blood cell count; PSM, propensity score matching.

SUPPLEMENTARY FIGURE 3. Survival Disparity by Bacterial Infection Status in ACLF Patients. Kaplan–Meier analysis revealed significantly shorter 90-day survival in ACLF patients with BI versus their NO BI counterparts (40% vs. 70%; log-rank P<0.001). BI, bacterial infection.

UPPLEMENTAL TABLE LEGENDS

SUPPLEMENTARY TABLE 1. Baseline Characteristics of Patients from PCT-included and NO PCT-included Cohorts.

**Variables**	**NO PCT-included（n=32）**	**PCT-included（n=136）**	***P value***
Sex, Male	22 (68.8%)	112 (82.4%)	0.085
Age(years)	54± 14	53± 12	0.528
Etiology			0.895
HBV	21 (65.6%)	82 (60.3%)	
Alcohol	5 (15.6%)	28 (20.6%)	
Alcohol+HBV	2 (6.2%)	11 (8.1%)	
Others	4 (12.5%)	15 (11%)	
Cirrhosis	22 (68.8%)	93 (68.4%)	0.968
MELD score	22 (19, 25)	24 (20, 28)	0.030*
MELD-Na score	23(19, 25)	26 (21, 33)	0.023*
Ascites	21 (65.6%)	91 (66.9%)	0.890

Variables	NO PCT-included（n=32）	PCT-included（n=136）	*P value*
HE	4 (12.5%)	25 (18.4%)	0.428
GI bleeding	0 (0%)	8 (5.9%)	0.345
AKI	6 (18.8%)	42 (30.9%)	0.172
Total bilirubin (μmol/L)	186.1 (131.3, 320.2)	220.1 (116.6, 396.8)	0.465
Albumin(g/L)	30.9 ± 5.5	30.0 ± 5.3	0.390
Creatinine (μmol/L)	67.0 (56.8, 88.8)	78.0 (61.7, 109.5)	0.042*
Sodium(mmol/L)	137.0 (133.7, 137.8)	135.7 (131.6, 138.0)	0.508
INR	1.6 (1.5, 1.8)	1.8 (1.6, 2.2)	0.002**
WBC ($\times 10^9$/L)	5.28(4.03, 6.19)	7.12 (4.73, 10.62)	0.003**
Platelet count ($\times 10^9$/L)	87 (66, 131)	87 (54, 138)	0.982
CRP(mg/L)	18.8 (10.6, 31.4)	20.4 (9.0, 35.9)	0.605
DLL1(ng/mL)	2.86(1.96, 3.18)	2.95 (1.92, 4.07)	0.344

Notes: *$P<0.05$, **$P<0.01$, ***$P<0.001$. Alcohol+HBV indicates patients with both alcohol-related liver disease and hepatitis B virus infection.MELD: Model for End-Stage Liver Disease; MELD-Na: Model for End-Stage Liver Disease sodium; HBV:Hepatitis B virus; HE: hepatic encephalopathy; GI: gastrointestinal; AKI: acute kidney injury; INR: international normalized ratio; WBC: white blood cell; CRP: C-reactive protein; PCT: procalcitonin; DLL1: Delta-like ligand.

SUPPLEMENTARY TABLE 2. Comparative Clinical Profiles Stratified by Bacterial Infection Status in ACLF after PSM

Variables	NO BI(n=49)	BI(n=49)	*P value*
Sex, Male	41 (83.7%)	36 (73.5%)	0.218

**Variables**	**NO BI(n=49)**	**BI(n=49)**	***P value***
Age(years)	52 ± 14	53 ± 12	0.646
Etiology			0.302
HBV	34 (69.4%)	25 (51%)	
Alcohol	8 (16.3%)	12 (24.5%)	
Alcohol+HBV	3 (6.1%)	4 (8.2%)	
Others	4 (8.2%)	8 (16.3%)	
Cirrhosis	24 (49%)	43 (87.8%)	< 0.001***
MELD-Na score	24 (20, 29)	24 (20, 27)	0.771
Ascites	25 (51%)	38 (77.6%)	0.006**
HE	4 (8.2%)	7 (14.3%)	0.337
GI bleeding	0 (0%)	3 (6.1%)	0.241
AKI	10 (20.4%)	10 (20.4%)	1.000
Total bilirubin (μmol/L)	219.7 (124.8, 378.9)	206.0 (107.8, 354.3)	0.768
Albumin(g/L)	32.6± 5.6	29.4± 4.4	0.003**
Creatinine (μmol/L)	69.0 (60.0, 84.5)	73.0 (62.0, 87.0)	0.334
Sodium(mmol/L)	137.2(133.8, 138.2)	135.3 (132.8, 137.0)	0.119
INR	1.7 (1.5, 1.9)	1.7 (1.5, 1.9)	0.738
WBC (×109/L)	6.00 (4.54, 8.58)	7.60 (4.93, 12.36)	0.100
Platelet count ($\times 10^9$/L)	101 (72, 143)	75 (54, 121)	0.069
CRP(mg/L)	18.2 (8.5, 26.2)	25.5 (14.7, 39.1)	0.010*
DLL1(ng/mL)	2.10 (1.52, 3.01)	3.53 (2.72, 4.11)	< 0.001***

Notes: *P<0.05, **P<0.01, ***P<0.001.Alcohol+HBV indicates patients with both alcohol-related liver disease and hepatitis B virus infection.ACLF: Acute-on-Chronic Liver Failure; PSM: Propensity Score Matching; BI: bacterial infection; NO BI: no bacterial infection; HBV: Hepatitis B virus; MELD-Na: Model for End-Stage Liver Disease sodium;HE: hepatic encephalopathy; GI: gastrointestinal; AKI: acute kidney injury; INR: international normalized ratio; WBC: white blood cell; CRP: C-reactive protein; DLL1: Delta-like ligand.

SUPPLEMENTARY TABLE 3. Independent Predictors of Bacterial Infection in Patients with ACLF after PSM

**Variables**	**Multivariate analysis**	
	**Odds Ratio (95% CI)**	***P value***
Cirrhosis	7.004 (1.737 – 28.245)	0.006**
DLL1(ng/mL)	1.722 (1.086 – 2.730)	0.021*

Notes: *P<0.05, **P<0.01, ***P<0.001. ACLF: Acute-on-Chronic Liver Failure; PSM: Propensity Score Matching; DLL1: Delta-like ligand.

SUPPLEMENTARY TABLE 4. Correlation analysis between DLL1 and clinical parameters.

**Variables**	***correlation_spearman***	***padj_spearman***
DLL1	1	0***
MELD score	0.404	0.000***
MELD-Na score	0.392	0.000***
Total bilirubin	0.173	0.033*
Albumin	0.252	0.002**
Creatinine	0.407	0.000***
Sodium	0.156	0.051
INR	0.214	0.008**

Variables	*correlation_spearman*	*padj_spearman*
WBC	0.223	0.006**
PLT	0.117	0.133
CRP	0.398	0.000***
Platelet	0.385	0.000***

Notes: *P<0.05, **P<0.01, ***P<0.001.DLL1: Delta-like ligand 1; MELD: Model for End-Stage Liver Disease; MELD-Na: Model for End-Stage Liver Disease sodium; INR: international normalized ratio; WBC: white blood cell; CRP: C-reactive protein; PCT: procalcitonin.
